# Occupational stress and burnout experience among healthcare workers compounded by the COVID-19 pandemic in Africa: a scoping review protocol

**DOI:** 10.1186/s13643-023-02200-w

**Published:** 2023-03-08

**Authors:** George Agyemang, Yaa Bema, Deborah Aba Eturu, Vitalis Bawontuo, Desmond Kuupiel

**Affiliations:** 1 Faculty of Health and Allied Sciences, Catholic University College of Ghana, Fiapre, Sunyani, Ghana; 2 Department of Health Services Management and Administration, School of Business, SD Dombo University of Business and Integrated Development Studies (SDD-UBIDS), Wa, Ghana; 3 Faculty of Health Sciences, Durban University of Technology, Ritson Campus, Durban, 4001 South Africa; 4 Department of Public Health Medicine, School of Nursing and Public Health, University of KwaZulu-Natal, Durban, 4001 South Africa

**Keywords:** Occupational stress, Burnout, Healthcare workers, COVID-19, Africa, Scoping review

## Abstract

**Background:**

The emergence of coronavirus disease 2019 (COVID-19) pandemic has brought an unprecedented burden on health systems and personnel globally. This pandemic potentially can result in increased frequency of stress and burnout experienced among healthcare workers (HCWs), especially in lower-and-middle-income countries with inadequate health professionals, yet little is known about their experience. This study aims to describe the range of research evidence on occupational stress and/burnout among HCWs compounded by the COVID-19 pandemic in Africa, as well as identify research gaps for further investigations to inform health policy decisions towards stress and/burnout reduction in this era and when a future pandemic occurs.

**Method:**

Arksey and O’Malley’s methodological framework will be used to guide this scoping review. Literature searches will be conducted in PubMed, CINAHL, SCOPUS, Web of Science, Science Direct, and Google Scholar for relevant articles published in any language from January 2020 to the last search date. The literature search strategy will include keywords and Boolean and medical subject heading terms. This study will include peer-reviewed papers about Africa, with a focus on stress and burnout among HCWs in the COVID-19 era. In addition to the database searches, we will manually search the reference list of included articles as well as the World Health Organization’s website for relevant papers. Using the inclusion criteria as a guide, two reviewers will independently screen the abstracts and full-text articles. A narrative synthesis will be conducted, and a summary of the findings reported.

**Discussion:**

This study will highlight the range of literature HCWs’ experience of stress and/or burnout including the prevalence, associated factors, interventions/coping strategies, and reported effects on healthcare services during the COVID-19 era in Africa. This study’s findings will be relevant to inform healthcare managers plan to mitigate stress and/or burnout as well prepare for such future pandemics. This study’s findings will be disseminated in a peer-review journal, scientific conference, academic and research platforms, and social media.

**Supplementary Information:**

The online version contains supplementary material available at 10.1186/s13643-023-02200-w.

## Background

Stress is described as unforeseen loads and pressures which distinguish between the individual’s knowledge and forecast and prevent his/her capacity to cope [1]. Stress generally may result from negative experiences related to anything from daily hassles, relationship issues, pressures at work, health concerns, financial challenges, and debilitating phobias [1]. Occupational stress is a known health risk for a range of behavioral, psychological, diseases, and medical disorders [2]. Occupational stress most often results from inadequate pay, inequality at work, too much workload, inadequate human resource capacity, poor recognition and promotion, time pressure, job insecurity, and lack of management support among others [3]. Occupational stress is estimated to range between 9% and 68% globally, with Sub-Saharan Africa being among the worst affected [4]. All workers potentially are at risk of occupational stress; however, with the current situation of the coronavirus disease 2019 (COVID-19), pandemic healthcare workers (HCWs) especially those at the forefront such as doctors, nurses, biomedical scientists, and pharmacists are potentially more at risk of occupational stress.

COVID-19, a disease caused by SARS-CoV-2, at the time of writing this protocol has infected more than 110,384,747 people with about 2.2% deaths globally [5]. Among HCWs, Amnesty International reported that over 7000 health workers have died worldwide due to COVID-19 infection so far [6]. In the USA alone, over 250,000 health workers were reported to have been infected, and nearly 1000 deaths have occurred [6]. HCWs are at the forefront of providing essential services to patients and may be experiencing additional occupational stress, burnout, and other psychological issues such as depression and anxiety due to the increasing burden of COVID-19 cases [7].

As a result of the additional occupational stress and burnout HCWs are potentially experiencing in this era of COVID-19 [7], the quality of care healthcare delivery or services may be affected if left unchecked, especially in lower-and-middle-income countries including African countries with inadequate healthcare professionals. To this end, studies synthesizing literature and describing the evidence would be useful to inform management decisions and prioritize future research toward preventing or reducing occupational stress and burnout among HCWs in this COVID-19 era. A scoping review is essential in mapping concepts or various evidence that are centered around a research field of interest, as well as identifying literature gaps to prioritize research [8].

Although Robertson and Colleagues conducted a scoping review aimed to inform provincial guidelines on the mental health of HCWs during the COVID-19 outbreak in South Africa [9], the literature suggests limited scoping reviews focusing on occupational stress and burnout among HCWs in this COVID-19 era to best of our knowledge. Knowledge of occupational stress and burnout among HCWs in this COVID-19 era is essential to all healthcare managers across the globe especially those in countries with weak systems, but due to lack of funding, this study will be limited to African countries only. This study aims to describe the range of research evidence on occupational stress and/burnout among HCWs compounded by the COVID-19 pandemic in Africa. This study will hope to report the prevalence, associated factors, interventions/coping strategies, and reported effects of occupational stress and burnout among Africa’s HCWs on healthcare delivery in this COVID-19 era. This study also hopes to identify research gaps for further investigations to inform health policy decisions toward stress and/or burnout reduction in this era and when a future pandemic occurs.

## Methods

The preferred reporting items for systematic reviews and meta-analysis extension for protocols (PRISMA-P) guideline was adopted to develop this study protocol (Supplementary file 1). This scoping review will be conducted in line with Arksey and O’Malley’s framework [10] incorporating the Levac and Colleagues’ [11] recommendations outlined below.

### Identifying the research question

The main review ‘question will be the following: to date, what research evidence on occupational stress and/or burnout among frontline healthcare workers compounded by the COVID-19 pandemic in Africa exists? Table 1 illustrates the population, concept, and context (PCC) framework used to check the suitability of this review question.

**Table 1:** The PCC defining the suitability of the scoping review question

Population	**Healthcare workers.** This will include all frontline healthcare workers such as doctors, nurses, midwives, biomedical scientists (medical laboratory professionals), and pharmacists
Concept	**Occupational stress**: This refers to the ongoing or progressing stress a healthcare worker experiences due to the responsibilities, conditions, environment, or other pressures of the workplace due to the COVID-19 pandemic **Burnout:** This refers to a state of emotional, physical, and mental exhaustion caused by excessive and prolonged stress [12]
Context	Africa: This will include all African countries that recorded COVID-19 cases

The sub-review questions for this proposed study will be as follows:

- What evidence exists on the prevalence of occupational stress and/or burnout among frontline HCWs (doctors, nurses, midwives, biomedical scientists, and pharmacists) compounded by the COVID-19 pandemic in Africa?
- What evidence exists on factors associated with occupational stress and/or burnout among HCWs compounded by the COVID-19 pandemic in Africa?
- What interventions or coping strategies exist for HCWs experiencing stress and burnout compounded by the COVID-19 pandemic in Africa?
- What evidence exists on the effect of occupational stress and/or burnout experienced by HCWs compounded by the COVID-19 pandemic on healthcare services?

### Identifying relevant studies

The search will be carried out in the following electronic databases: PubMed, CINAHL, SCOPUS, Web of Science, Science Direct, and Google Scholar, for relevant peer-review papers and grey literature to answer the research question. In collaboration with an expert librarian, a search strategy will be developed using the following keywords (“health worker”, “health care worker”, “HCWs”, “health professional”, “medical personnel”, “health provider”, “doctor”, “nurse”, “biomedical scientist”, “medical laboratory personnel”, “occupational stress”, “professional stress”, “burnout”, “COVID-19”, “coronavirus”, “2019-ncov”, “sars-cov-2”, “cov-19”, and “Africa). Boolean terms (AND/OR), as well as Medical Subject Heading (MeSH) terms, will be included to optimize the search in the databases. Language, publication type, and study design limitations will be removed; however, the search will be limited to humans and date (from January 2020 to the search date in 2023). Table 2 illustrates a pilot search strategy conducted in PubMed and a search documentation plan. In addition to the electronic databases, the reference list of the included studies will be manually searched as well as the WHO website for relevant studies and grey literature. George Agyeman (AG) who is the principal researcher will conduct the electronic database search and title screening simultaneously. All the titles that meet this review eligibility criterion will be imported into Mendeley Desktop Library which will be used to manage all citations for this study.

**Table 2:** A pilot search strategy conducted in PubMed

Date	Database	Keywords	Search results
16 February 2023	PubMed	(“occupational stress”[MeSH Terms] OR (“occupational”[All Fields] AND “stress”[All Fields]) OR “occupational stress”[All Fields] OR (“burnout s”[All Fields] OR “burnout, psychological”[MeSH Terms] OR (“burnout”[All Fields] AND “psychological”[All Fields]) OR “psychological burnout”[All Fields] OR “burnout”[All Fields] OR “burnouts”[All Fields])) AND (“health personnel”[MeSH Terms] OR (“health”[All Fields] AND “personnel”[All Fields]) OR “health personnel”[All Fields] OR (“healthcare”[All Fields] AND “workers”[All Fields]) OR “healthcare workers”[All Fields]) AND (“covid 19”[MeSH Terms] OR “covid 19”[All Fields] OR “covid 19 pandemic”[All Fields])	1935

### Study eligibility criteria and selection

#### Eligibility criteria

##### Inclusion criteria

The study will include all publication types in any language that focuses on Africa. Also, this study will include articles that:

- Involved frontline healthcare workers (doctors, nurses, biomedical scientists, and pharmacists),
- Focus on occupational stress and/or burnout compounded by the COVID-19 pandemic,
- Report on factors associated with occupational stress and/or burnout,
- Report on occupational stress and/or burnout interventions or coping strategies, and
- Report evidence on the effect of occupational stress and/or burnout experienced by frontline HCWs compounded by the COVID-19 pandemic on healthcare services.
- Study designs (qualitative, quantitative, mixed methods, and systematic reviews).

##### Exclusion criteria

The study will exclude the following:

- Articles focused on other HCWs such as supporting staff
- Publications before January 2020,
- Research that did not involve occupational stress and/burnout and COVID-19,
- Articles that focus on other professionals such as teachers,
- Articles focusing on patients, and
- Grey literature, editorials, expert opinions, and others alike.

### Study selection

**Fig. 1:**
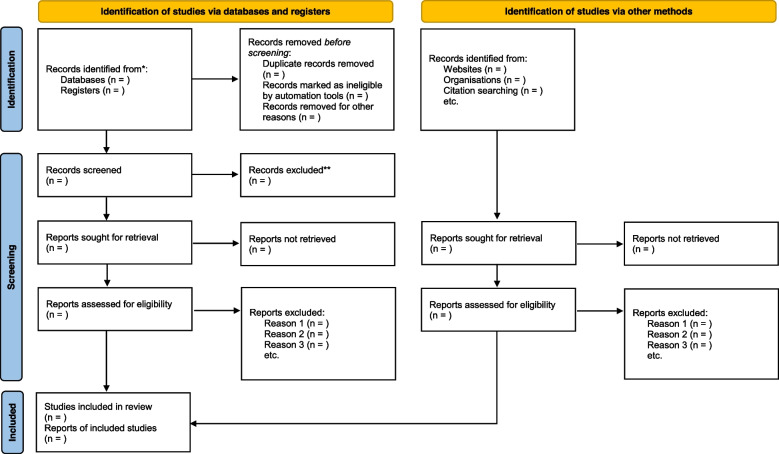
PRISMA flow diagram

Before the abstract screening stage, all duplicate articles in the Mendeley Desktop Library will be removed. A screening tool will be developed a priori, pilot-tested, and amended appropriately. The abstract screening will be conducted independently by AG and YB using this study’s eligibility criteria with guidance from the project supervisor (DK). Any discrepancy at the abstract screening stage will be discussed by all the review team members until a consensus is reached. The full text articles of all abstracts that meet the inclusion criteria will be sought and compiled for screening. Efforts will be made to obtain the full-text articles of all closed access publications by utilizing the Catholic University and Durban University of Technology library services. Emails will also be sent to the original authors of such articles requesting the full text. At the full text article screening stage, AG and YB will again apply this study’s eligibility criteria to “include” and “exclude” appropriately. A third reviewer (DE) will be engaged to resolve any discrepancy between AG and YB during the full text screening phase. To ensure accountability of the selection process, this study will employ the 2020 PRISMA flow diagram to present the screening results (Fig. 1).

### Charting the data

Two reviews (AG, YB) will extract the data from the included studies independently using a piloted tested form. A third reviewer (DE) will resolve any discrepancies that arise between AG and YB. We will extract the characteristics of the included studies (author(s), publication year, study geographical setting (country), study population type (doctors, nurses, biomedical scientists, or pharmacists), gender of study participants, and study setting (hospital or clinic). We will also extract results relating to the prevalence of occupational stress and/or burnout, factors associated with occupational stress and/or burnout, interventions or coping strategies that exist for HCWs experiencing stress and burnout due to COVID-19, and the effect of occupational stress and/or burnout experienced by HCWs due to COVID-19 pandemic on healthcare services. All other related knowledge or pieces of evidence present in the conclusion and/or recommendations of the included articles will be extracted to answer this scoping review question.

### Collating summarizing and reporting the results

A narrative synthesis will be used to summarize all relevant data into themes and sub-themes to answer the review question. The finding will be grouped into four themes (prevalence, contributing/associated factors, interventions/coping strategies, and reported effect) and reported. Other relevant emerging themes or sub-themes will be reported. Tables and figures/maps will also be used to present the characteristics of the included studies, study results, and study findings that were appropriate. The reporting of this study will follow the PRISMA extension for scoping review (ScR) [13].

## Discussion

The COVID-19 pandemic continues to have a profound impact on the health system and HCWs. Occupational stress forms a significant component of overall mental well-being problems, economic shifts, and related financial burdens. Knowledge about occupational stress and burnout among HCWs plays a crucial role in preventing severe consequences on already challenging health systems. The results of this study will be published in a peer-review journal and on social media. The results will also be disseminated via conferences and discussions with relevant authorities as well as discussions in electronic media such as radio and television stations. It is expected that the results of this proposed scoping review will help to identify literature gaps to guide future research such as systematic reviews and primary studies to address occupation stress and burnout among HCWs in the African context. This scoping review will also provide evidence-based information to health managers for planning to mitigate any potential negative implications on the health system arising from occupational stress and/burnout experienced by HCWs during COVID-19.

## Supplementary Information

**Additional file 1: Supplementary file 1.** PRISMA-P 2015 Checklist.

## Data Availability

All papers or documents used for this review protocol have been added to the reference list.

## Abbreviations

HCWs: Healthcare workers
COVID-19: Coronavirus diseases 2019
